# Lactoferrin and Thioredoxin in Rheumatoid Arthritis Are Associated with Fibrinogen but Not with Other Acute Phase Proteins

**DOI:** 10.3390/ijms26178211

**Published:** 2025-08-24

**Authors:** Ginka Delcheva, Katya Stefanova, Pavel Selimov, Teodora Stankova

**Affiliations:** 1 Department of Medical Biochemistry, Faculty of Pharmacy, Medical University of Plovdiv, 15A Vasil Aprilov Blvd., 4002 Plovdiv, Bulgaria; katya.stefanova@mu-plovdiv.bg (K.S.); teodora.stankova@mu-plovdiv.bg (T.S.); 2 Department of Propaedeutic of Internal Diseases, Faculty of Medicine, Medical University of Plovdiv, 15A Vasil Aprilov Blvd., 4002 Plovdiv, Bulgaria; pavel.selimov@mu-plovdiv.bg

**Keywords:** lactoferrin, thioredoxin, fibrinogen, ferritin, CRP, rheumatoid arthritis, oxidative stress, inflammation

## Abstract

Rheumatoid arthritis (RA) is one of the most common chronic autoimmune diseases which global prevalence is approximately 0.3–2%. Numerous studies provide evidence that the elevated levels of reactive oxygen species (ROS) contribute to the pathogenesis and progression of RA. In response to redox imbalance, several intrinsic antioxidant defence mechanisms are activated to counteract oxidative stress and scavenge ROS. The aim of the present study is to analyse whether the levels of lactoferrin and thioredoxin, two proteins which are part of the antioxidant defence of the body, are associated with fibrinogen and other acute phase proteins such as CRP and ferritin in RA. Serum lactoferrin, thioredoxin, ferritin, and CRP levels were measured using ELISA. Significant positive correlations of lactoferrin and thioredoxin with fibrinogen were observed in RA patients, r = 0.394, *p* < 0.0001 and r = 0.410, *p* = 0.002, respectively. These positive correlations were also observed in females, r = 0.375, *p* < 0.0001 and r = 0.447, *p* = 0.001, in the subgroup of patients with DAS28 < 5.1, r = 0.689, *p* < 0.0001 and r = 0.604, *p* = 0.001 and in the subgroup of patients with normal CRP, r = 0.488, *p* < 0.0001 and r = 0.414, *p* = 0.005, respectively. These findings help clarify the pathogenetic interplay between oxidative stress, inflammation, and coagulation in RA and indicate the need for further studies to elucidate the potential of lactoferrin and thioredoxin as biomarkers that capture pathological disease changes.

## 1. Introduction

Rheumatoid arthritis is one of the most common chronic autoimmune diseases, with a global prevalence estimated at approximately 0.3–2%. The disease is currently incurable and affects women three times more often than men, with a peak onset around the age of 50 [1,2]. There is evidence that genetic and environmental factors—including smoking and immune dysregulation—may contribute to the development of RA [3]. The disease can lead to progressive disability, premature death, and significant socioeconomic burden. Clinical symptoms typically include joint pain, swelling, and stiffness, most often affecting both small and large joints, ultimately leading to cartilage destruction and bone erosion [4,5].

The pathogenesis of RA is complex and progresses through several stages before clinical manifestations become evident. The earliest phase of the disease involves genetic susceptibility. Specific genetic factors, such as the presence of human leukocyte antigen (HLA) alleles—particularly HLA-DRB1—play a key role in the autoimmune response. Other genes implicated in RA predisposition include PTPN22 (protein tyrosine phosphatase non-receptor type 22), STAT4 (signal transducer and activator of transcription-4), and TRAF1-C5 (tumor necrosis factor receptor-associated factor 1), although their exact roles in autoimmunity remain incompletely understood. Additionally, epigenetic modifications, such as altered DNA methylation, have also been identified as important components of RA etiology [6]. Environmental triggers—including smoking, infections, air pollutants, and diet—can initiate immune responses in genetically predisposed individuals, leading to the production of RA-specific autoantibodies. These include rheumatoid factors (RF), which are anti-immunoglobulin G (IgG) antibodies, and anti-citrullinated protein antibodies (ACPA), which can be elevated several years before the onset of clinical symptoms [6,7]. The amplification of the immune response results in infiltration of synovial tissue by dendritic cells, T cells, B cells, and macrophages, as well as the activation of synovial fibroblasts. This leads to synovial hyperplasia and angiogenesis [6,8]. Infiltrating immune cells and resident synovial cells produce pro-inflammatory cytokines such as tumor necrosis factor (TNF), interleukin-6 (IL-6), and interleukin-1 (IL-1), which are key drivers of RA pathogenesis. These cytokines promote synovial inflammation, joint damage, and bone erosion, contributing to early symptoms such as morning stiffness, mild arthralgia, and fatigue [6,8]. RA typically has an insidious onset, beginning with pain and swelling in one or more joints. It commonly affects the wrists and small joints of the hands and feet, although larger joints can also be involved [7]. The diagnosis and classification of RA are guided by the 2010 criteria established by the American College of Rheumatology (ACR) and the European League Against Rheumatism (EULAR). These criteria include parameters such as the number and distribution of swollen or tender joints, the presence and levels of RA-specific autoantibodies (e.g., RF and ACPA), levels of acute-phase reactants (e.g., CRP and ESR), and the duration of symptoms [7,9].

Although RA remains incurable, advancements in pharmacological therapy have significantly improved patient quality of life by alleviating symptoms, slowing disease progression, and achieving remission in some cases. Two primary treatment strategies are employed: a symptomatic approach using nonsteroidal anti-inflammatory drugs (NSAIDs) and glucocorticoids, and a disease-targeted approach using disease-modifying antirheumatic drugs (DMARDs). DMARDs can be categorized as conventional synthetic, targeted synthetic, or biological agents. In recent years, researchers have increasingly focused on identifying bioactive phytocompounds with anti-inflammatory and antioxidant properties. These natural compounds and their derivatives may act on key proteins and signaling pathways involved in RA pathogenesis and are being explored as potential adjuvants in RA treatment [10].

**Figure 1:**
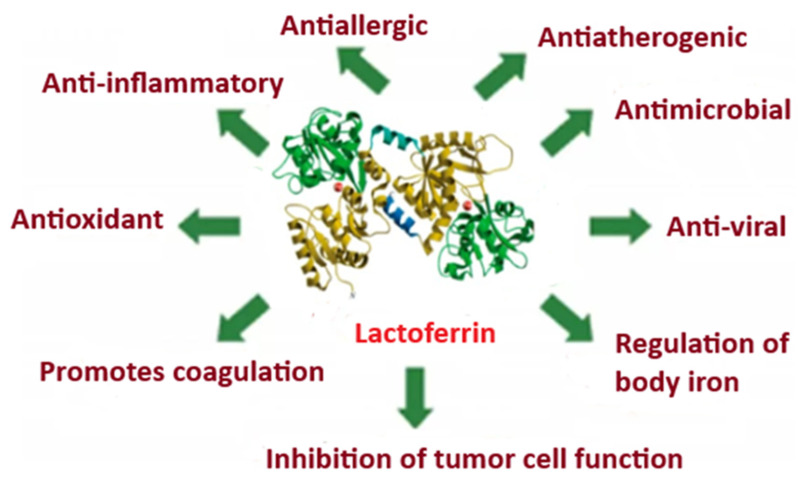
A schematic representation illustrating some of the known effects of lactoferrin—adapted from [15].

Lactoferrin (Lf) is an iron-binding glycoprotein composed of 691 amino acids, with a molecular mass of approximately 77 kDa and containing 16 disulfide bonds. It is found in colostrum, breast milk, digestive secretions, saliva, nasal secretions, tears, and semen. A major portion of circulating Lf in plasma originates from neutrophils, where it is stored in granules [11,12,13,14]. The three-dimensional structure of diferric human lactoferrin and some of its known functions are shown in Figure 1.

Lf can also be consumed as a dietary supplement, offering potential immune support due to its antibacterial, antiviral, and antioxidant properties [16]. Recent studies have shown that Lf can bind to certain receptors utilized by coronaviruses, thereby blocking viral entry into host cells. As such, Lf has been suggested as a potential preventive and therapeutic agent in COVID-19 [16].

Lactoferrin concentration in serum is strongly influenced by inflammatory processes associated with neutrophil degranulation, which leads to Lf secretion [17]. Neutrophils, the most abundant and shortest-lived leukocytes, are both granulocytes and phagocytes, typically surviving only 24–48 h in circulation before undergoing apoptosis [18]. Since delayed neutrophil apoptosis may contribute to increased tissue damage, chronic inflammation, and disease pathology, tight regulation of neutrophil lifespan is essential to prevent the development of chronic inflammatory conditions such as rheumatoid arthritis (RA) [18]. Hypoxic conditions in the synovial fluid of rheumatoid joints have been shown to delay neutrophil apoptosis and are proposed as a key factor prolonging neutrophil survival [19]. Previous studies have highlighted the importance of reactive oxygen species (ROS) in neutrophil apoptosis and have investigated the role of the iron-chelating protein lactoferrin in modulating this process within the synovial environment [20,21,22].

Thioredoxin (Trx) is a small, ubiquitous redox-active protein with a molecular mass of 12 kDa, involved in various cellular functions, including DNA synthesis, protection against oxidative stress, cell proliferation, and inhibition of apoptosis [23,24]. The thioredoxin system comprises thioredoxin, thioredoxin reductase (TrxR), and NADPH. Thioredoxin primarily functions by reducing disulfide bonds in target proteins and can convert cystine to cysteine, a precursor for glutathione (GSH) synthesis [25]. Although initially identified as an extracellular protein, Trx is now known to be distributed intracellularly, including in the cytoplasm, mitochondria, and nucleus. It plays a central role in maintaining redox homeostasis by transferring reducing equivalents to oxidized proteins, thereby restoring their function and protecting cells from oxidative damage [24,26].

Fibrinogen is a 340 kDa plasma glycoprotein composed of three polypeptide chains and secreted into the bloodstream. It is the final substrate in the blood coagulation cascade and also functions as an acute-phase protein whose expression is upregulated in response to inflammatory cytokines, particularly interleukin-6 (IL-6) [27,28]. Abnormal levels of circulating fibrinogen are associated with bleeding disorders and inflammatory diseases. During acute inflammation, fibrinogen levels can increase by up to three times. Therefore, investigating fibrinogen and its relationship with various biomarkers may provide insights into novel therapeutic approaches and contribute to the development of more effective clinical management strategies [27,29].

The aim of this study was to investigate whether serum levels of lactoferrin and thioredoxin—two key proteins involved in the body’s antioxidant defence—are associated with fibrinogen and other acute-phase proteins such as C-reactive protein (CRP) and ferritin in patients with RA.

## 2. Results

In our previous study we report the serum levels of Trx and Lf in patients with RA and their associations with rheumatoid factor and other disease markers such as anti-CCP antibodies, DAS28 and ESR [17].

In the present study we investigated the associations of the two proteins lactoferrin and thioredoxin with fibrinogen and other acute phase proteins such as ferritin and CRP in patients with RA. The patients with RA were distributed in two subgroups according to sex, CRP level, and DAS28 and the same correlations were also analysed.

The main demographic and clinical characteristics of the study RA patients as well as their medication regime are summarized in Table 1.

**Figure 2:**
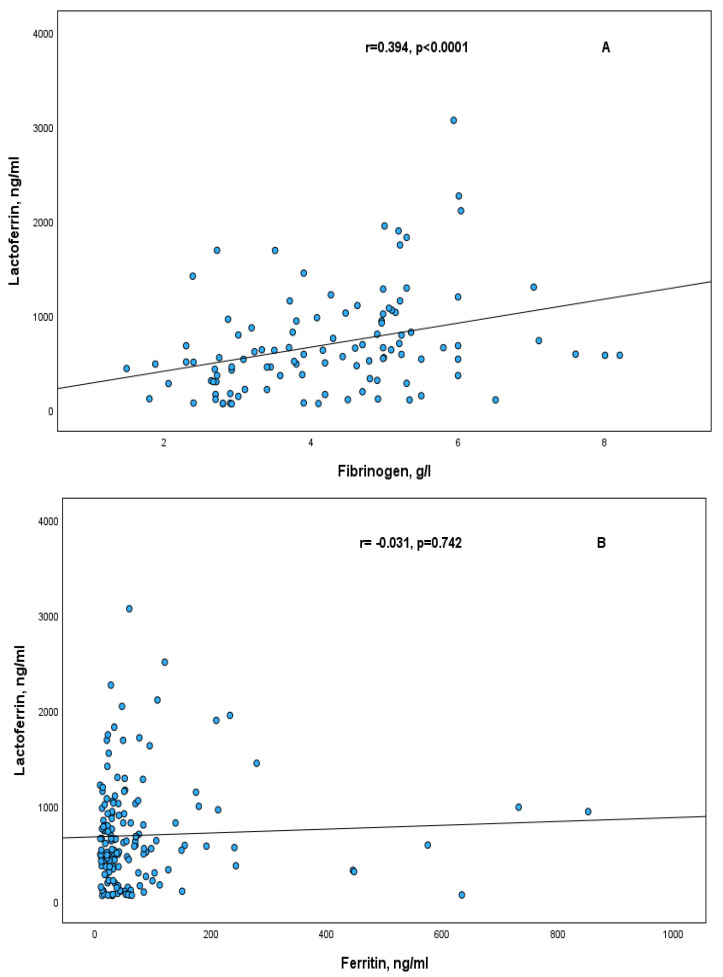

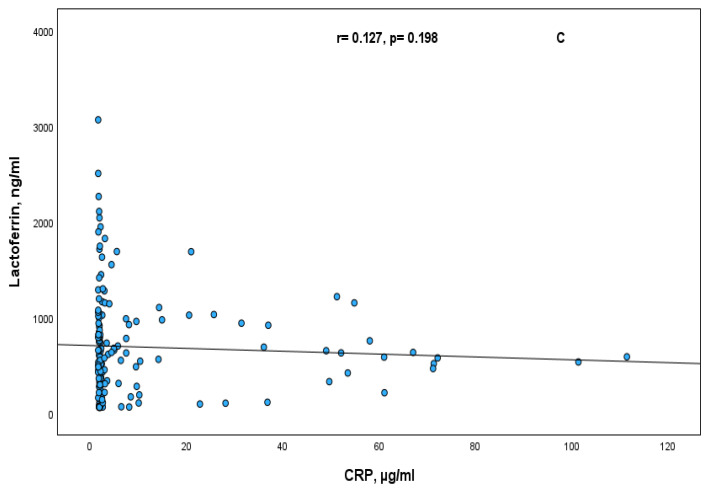
Correlation between serum lactoferrin and acute phase proteins in RA patients fibrinogen (**A**); ferritin (**B**); CRP (**C**).

**Figure 3:**
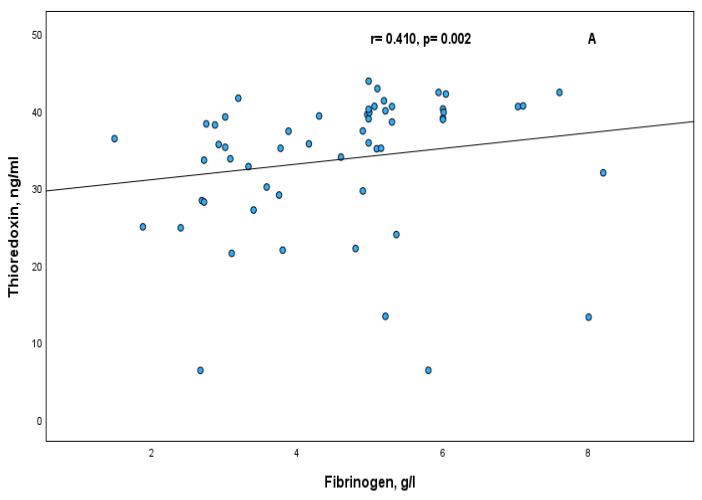

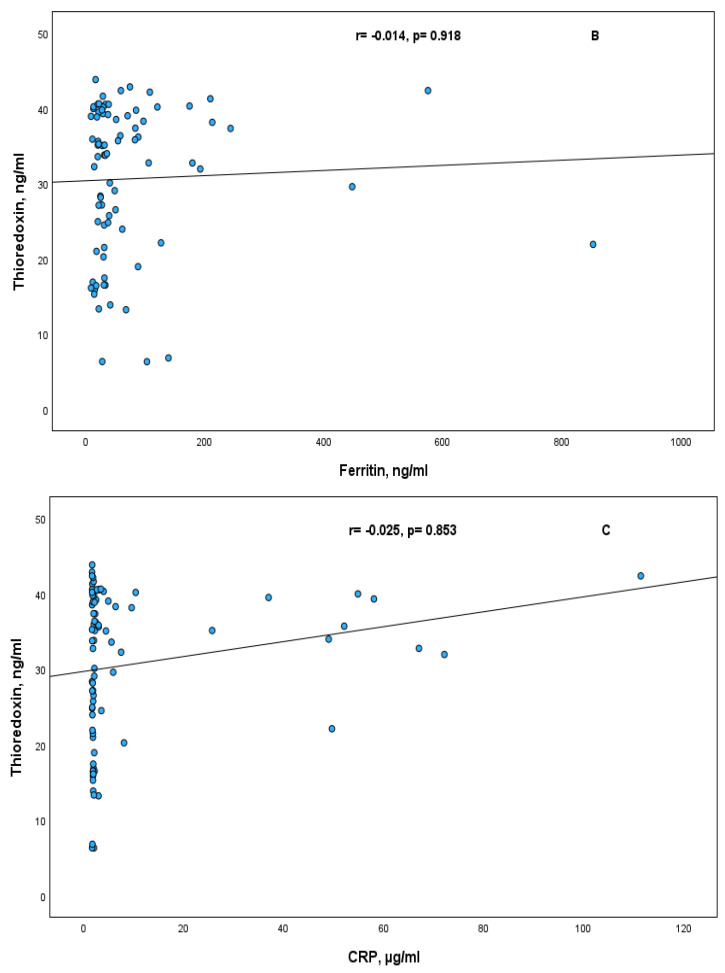
Correlation between serum thioredoxin and acute phase proteins in RA patients fibrinogen (**A**); ferritin (**B**); CRP (**C**).

Significant positive correlations of lactoferrin and thioredoxin with fibrinogen were observed in RA patients, r = 0.394, *p* < 0.0001 and r = 0.410, *p* = 0.002, respectively (Figure 2A and Figure 3A). These positive correlations were also observed in females of RA patients, in the subgroup of patients with moderate disease activity (DAS28 ≤ 5.1), and in the subgroup of patients with normal CRP level (Table 2).

The correlations of Lf and Trx in RA patients with other acute phase proteins such as ferritin and CRP were not statistically significant (Figure 2B,C and Figure 3B,C). The same results were observed also in the subgroups according to sex, CRP level and DAS28.

## 3. Discussion

In the present study, we investigated the associations of two antioxidant defence proteins—lactoferrin and thioredoxin—with fibrinogen and other acute-phase proteins, such as ferritin and C-reactive protein (CRP), in patients with rheumatoid arthritis. Our findings revealed that both Lf and Trx were positively correlated with fibrinogen but showed no significant correlation with ferritin or CRP. These results suggest that, in the context of chronic inflammation, oxidative stress may influence coagulation factors—particularly fibrinogen—highlighting its potential role in the pathophysiology of RA.

Fibrinogen is not only a central coagulation factor but also an acute-phase protein involved in inflammation. Fibrin accumulation within rheumatoid joints contributes to pannus formation and is considered pathogenic in RA [30]. Despite this, the role of circulating fibrinogen in RA patients remains insufficiently explored. One previous study reported impaired coagulation parameters and elevated fibrinogen levels in RA patients compared to healthy reference values. Importantly, six months of effective antirheumatic treatment was shown to restore haemostatic balance [31]. Coagulation activation during inflammation is driven by proinflammatory cytokines. Endothelial dysfunction induced by tumour necrosis factor (TNF) and platelet activation triggered by interleukin-6 (IL-6) lead to tissue factor expression on platelets and monocytes, enhancing thrombin generation and fibrin formation [31].

Lf has been described as a modulator of inflammation, oxidative stress, and apoptosis—mechanisms central to the progression of chronic diseases. It can regulate reactive oxygen species (ROS) production and elimination by modulating antioxidant enzymes, thus exerting potent anti-inflammatory effects [32]. Its concentration is strongly influenced by inflammatory processes that recruit neutrophils, which release Lf from secondary granules upon degranulation [17,32,33]. Neutrophils are the predominant cell type in the synovial fluid of RA joints, and Lf has been proposed as a marker of neutrophil activation [17]. In our previous study, we reported elevated serum Lf levels in RA patients, which were negatively correlated with rheumatoid factor [17]. In the current study, we found that serum Lf levels were positively correlated with fibrinogen, but not with other inflammatory markers such as CRP or ferritin. This association was also observed in subgroups with moderate disease activity and in patients with normal CRP levels. A recent study by Zhuravleva and Gusev further supports the link between coagulation, inflammation, and immunity in RA, reporting a strong association between systemic disease activity and hypercoagulation, as evidenced by elevated D-dimer levels [34]. The authors explain this procoagulant shift by the inhibition of the anticoagulant pathway, due to increased production of plasminogen activator inhibitor-1 (PAI-1), induced by IL-6, TNF, and TGF-β. This phenomenon, described as “immunothrombosis” or “thromboinflammation”, reflects the dysregulation of hemostasis in chronic inflammatory diseases [35]. Our results align with this concept, suggesting that Lf, as a marker of neutrophil activation, may play a role in systemic inflammation and its connection to coagulation. This finding adds a novel perspective on inflammatory processes that may not be captured by commonly used markers such as CRP.

Previous studies have identified Trx as a potential biomarker of RA disease activity, reflecting increased oxidative stress [36]. Plasma Trx levels have been reported to be significantly higher in RA patients compared to healthy controls and positively correlated with CRP and disease activity. Moreover, serum Trx levels were shown to correlate with Trx concentrations in synovial fluid. However, our findings do not support this correlation. In the current study, serum Trx levels were significantly associated with fibrinogen, but not with CRP or disease activity. Other studies have demonstrated that Trx is highly expressed in synovial fluid and synovial tissue in RA and is found in leukocytes and the synovial lining layer, suggesting a protective role against oxidative stress and inflammation [37]. In our earlier work, we reported a strong positive correlation between Lf and Trx in RA patients—observed across both sexes, in patients with DAS28 < 5.1 and DAS28 > 5.1, and in those with normal CRP levels. Additionally, both Lf and Trx were negatively correlated with RF, further suggesting their involvement in the immune response [17]. These results imply that Lf and Trx levels may rise simultaneously during disease progression, reflecting pathological changes in RA.

Other authors have emphasized the role of oxidative stress in enhancing protein citrullination, a post-translational modification catalyzed by peptidylarginine deiminases (PADs). This process is implicated in RA pathogenesis through the generation of anti-citrullinated protein antibodies (ACPA) [38]. ACPAs are associated with more severe disease and contribute to osteoclastogenesis and bone destruction [39]. Recent studies suggest that Trx, as a regulator of redox balance, may influence PAD activity through non-covalent interactions, linking it to protein citrullination. PADs target proteins such as fibrinogen, fibronectin, collagen, and enolase [40]. Among these, fibrinogen is a major autoantigen in the ACPA response. Complexes of citrullinated fibrinogen and ACPA present in the RA synovium can stimulate macrophage activation via TLR4 and Fc receptors, promoting the production of proinflammatory cytokines such as TNF, IL-1β, IL-6, and IL-8 [39]. Furthermore, fibrinogen interacts with complement receptors, and in its native form, inhibits osteoclastogenesis. However, citrullination alters fibrinogen’s structure and abolishes its osteoprotective function, contributing to bone erosion in RA [40]. Synovial fluid from inflamed RA joints also contains high levels of fibrinogen-derived peptides, including citrullinated fibrinopeptides, which may further enhance immune activation [41]. Given these findings, further research is needed to clarify fibrinogen’s role in RA pathophysiology, particularly its involvement in autoimmunity and inflammation through citrullination.

The interplay between coagulation and systemic inflammation in RA has been extensively discussed. It is now recognized that hypercoagulability is a hallmark of RA [42]. A large meta-analysis including over 14,000 RA cases confirmed a causal association between RA and seven coagulation factors, identifying a strong positive correlation between fibrinogen and RA risk [43]. These data emphasize the importance of studying the relationship between inflammatory markers, oxidative stress, and coagulation factors, which may lead to the identification of new therapeutic targets and more effective disease management strategies.

Several limitations of the study must be acknowledged. First, while the overall sample size was adequate, some subgroups were relatively small, limiting the statistical power of subgroup analyses. Second, the study was based on serum and plasma samples only; no data was available from synovial fluid, which may have provided additional insights. Third, pharmacological treatments received by some patients could have influenced the measured parameters. Lastly, the study cohort included a higher proportion of women, so future studies should aim for a more balanced sex distribution to ensure broader applicability of the findings.

## 4. Materials and Methods

The study population consisted of 114 patients with RA admitted to the Rheumatology departments of the University hospitals in Medical University of Plovdiv. All patients were diagnosed as having RA according to EULAR (European Alliance of Associations for Rheumatology) 2010 criteria. All patients were receiving NSAIDs and additional therapy as follows: DMARDs, DMARDs and corticosteroids, biological agents, DMARDs, and corticosteroids, corticosteroids, biological agents, and DMARDs, biological agents and corticosteroids. Patients with RA were distributed in two subgroups according to sex, CRP level and DAS28. CRP value 8 µg/mL and DAS28 of 5.1 were used as a cut-off point. Patients in the group with DAS28 < 5.1 had moderate disease activity and patients with DAS28 > 5.1 had high disease activity.

**Figure 4:**
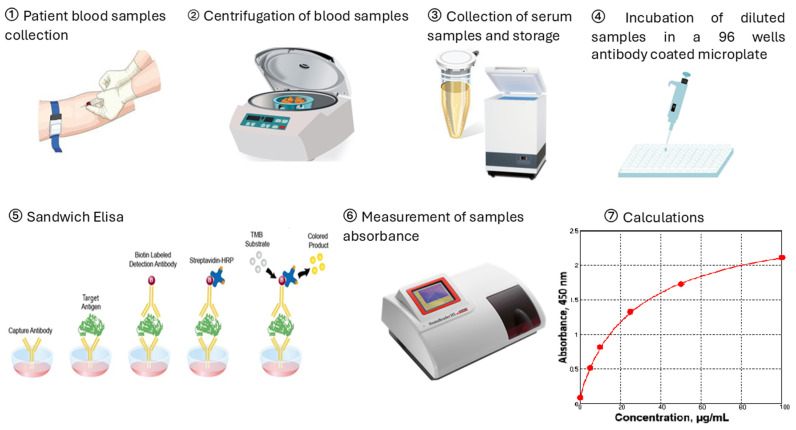
Schematic diagram of sample collection and experimental methods. Figure created with Paint 3D.

Blood was collected in monovettes without anticoagulant. Thirty minutes after blood collection, the tubes were centrifuged at 3000× *g* for 10 min. Serum was stored at −80 °C prior analysis. Serum concentrations of lactoferrin, ng/mL and thioredoxin 1, ng/mL were determined using commercially available ELISA kits (MyBioSource, San Diego, CA, USA and AbFrontier, Seoul, Republic of Korea). Serum concentrations of CRP, µg/mL and ferritin, ng/mL were determined with ELISA kits (BioVendor—Laboratornimedicina, Brno, Czech Republic). The measurements were performed on ELISA reader HumaReader HS, HUMAN (Wiesbaden, Germany). The experimental protocol is illustrated in Figure 4. Fibrinogen levels, g/L were measured by the Clauss fibrinogen assay in platelet-poor plasma at standard conditions. The analysis is performed on an automated hematology analyzer SYSMEX 2000i (Sysmex Corporation, Kobe, Japan).

Statistical analysis was performed using IBM SPSS Statistics software, version 17.0 and version 30.0. Normal distribution was assessed using the Kolmogorov-Smirnov test and continuous variables were expressed as mean ± SD or as median and 25th-percentile–75th-percentile. Correlations between data were evaluated by calculating Pearson’s or Spearman’s correlation coefficient depending on the distribution of the continuous variables. *p* < 0.05 was considered as statistically significant.

## 5. Conclusions

In conclusion, this study is the first to demonstrate that serum lactoferrin and thioredoxin levels are positively correlated with fibrinogen, but not with other acute-phase proteins such as ferritin and C-reactive protein (CRP) in patients with rheumatoid arthritis. These findings provide new insights into the pathogenetic interplay between oxidative stress, inflammation, and coagulation in RA. Moreover, they highlight the need for further research to explore the potential of lactoferrin and thioredoxin as biomarkers that reflect underlying pathological changes not captured by conventional inflammatory markers.

## Figures and Tables

**Table 1:** Demographic, clinical characteristics, and medication regime of the study RA patients.

Characteristics	
Mean age, years	58 ± 10 years
Sex (M/F), *n* (%)	16 (14%)/98 (86%)
DAS28	5.7 ± 1.2, *n* = 114
CRP, µg/mL	2.7 (1.9–10.3), *n* = 114
ESR, mm/h	36 (28–58), *n* = 114
Lactoferrin, ng/mL	579.6 (312.8–947.5)
Thioredoxin, ng/mL	36.4 (29.6–40.2)
Fibrinogen, g/L	4.25 ± 1.38, *n* = 108
RF, U/mL	56 (20–198), *n* = 107
Anti-CCP antibodies, U/mL	140 (54–246), *n* = 67
*Medication regime of the patients*	
NSAIDs and DMARDs	*n* = 83
NSAIDs, DMARDs, and corticosteroids	*n* = 10
NSAIDs, biological agents, DMARDs, and corticosteroids	*n* = 4
NSAIDs and corticosteroids	*n* = 2
NSAIDs, biological agents, and DMARDs	*n* = 11
NSAIDs, biological agents, and corticosteroids	*n* = 3

Data are presented as mean ± SD or median (25th–75th percentile).

**Table 2:** Correlations of lactoferrin and thioredoxin with fibrinogen in subgroups of RA patients according to sex, DAS28, and CRP level.

Parameters	r	*p*	r	*p*
	Males		Females	
Lactoferrin/Fibrinogen		NS	0.375	<0.0001
Thioredoxin/Fibrinogen		NS	0.447	0.001
	**DAS28 ≤ 5.1**		**DAS28 > 5.1**	
Lactoferrin/Fibrinogen	0.689	<0.0001		NS
Thioredoxin/Fibrinogen	0.604	0.001		NS
	**CRP ≤ 8**		**CRP > 8**	
Lactoferrin/Fibrinogen	0.488	<0.0001		NS
Thioredoxin/Fibrinogen	0.414	0.005		NS

## Data Availability

The data presented in this study are available upon reasonable request from the corresponding author.
